# PpTFDB: A pigeonpea transcription factor database for exploring functional genomics in legumes

**DOI:** 10.1371/journal.pone.0179736

**Published:** 2017-06-26

**Authors:** Akshay Singh, Ajay Kumar Sharma, Nagendra Kumar Singh, Tilak Raj Sharma

**Affiliations:** 1 National Research Centre on Plant Biotechnology, Pusa Campus, New Delhi, India; 2 Dr. A. P. J. Abdul Kalam Technical University, Lucknow, Uttar Pradesh, India; 3 Meerut Institute of Engineering and Technology, Meerut, Uttar Pradesh, India; National Institute for Plant Genome Research, INDIA

## Abstract

Pigeonpea (*Cajanus cajan L*.), a diploid legume crop, is a member of the tribe *Phaseoleae*. This tribe is descended from the millettioid (tropical) clade of the subfamily Papilionoideae, which includes many important legume crop species such as soybean (*Glycine max*), mung bean (*Vigna radiata*), cowpea (*Vigna ungiculata*), and common bean (*Phaseolus vulgaris*). It plays major role in food and nutritional security, being rich source of proteins, minerals and vitamins. We have developed a comprehensive Pigeonpea Transcription Factors Database (PpTFDB) that encompasses information about 1829 putative transcription factors (TFs) and their 55 TF families. PpTFDB provides a comprehensive information about each of the identified TFs that includes chromosomal location, protein physicochemical properties, sequence data, protein functional annotation, simple sequence repeats (SSRs) with primers derived from their motifs, orthology with related legume crops, and gene ontology (GO) assignment to respective TFs. (PpTFDB: http://14.139.229.199/PpTFDB/Home.aspx) is a freely available and user friendly web resource that facilitates users to retrieve the information of individual members of a TF family through a set of query interfaces including TF ID or protein functional annotation. In addition, users can also get the information by browsing interfaces, which include browsing by TF Categories and by, GO Categories. This PpTFDB will serve as a promising central resource for researchers as well as breeders who are working towards crop improvement of legume crops.

## Introduction

Pigeonpea [*Cajanus cajan* (L.) Millspaugh], a diploid legume crop (2n = 2x = 22), is a member of the tribe *Phaseoleae* with the estimated genome size of 858 Mbp. It is the main source of proteins, minerals and vitamins for more than a billion people in the developing world. In addition, this plant is not only useful as a source of nutrition for human consumption but their leaves, seed and pod husks are used as animal feed. Pigeonpea is unique among all the legume crops because it is a woody shrub, and its stem and branches are used for firewood, fencing, thatch and making baskets by the rural population [1, 2]. Therefore, cultivation of pigeonpea (*C*. *cajan*) is beneficial for both economic, health and environmental perspective. It is known for their high nitrogen fixation ability from the atmosphere with the help of symbiotic nitrogen-fixing bacteria (*Bradyrhizobium)*. Its nitrogen fixation ability reduces the need of synthetic crop fertilizers hence reduces cause of water pollution [3]. According to the Agricultural and Processed Food Products Export Development Authority (APEDA), 2, 51,644.32 MT of pulses has been exported by India to different countries of the world amounting to Rs. 1,603.22 crores during the year 2015–16. The other major exporting countries are Pakistan, Algeria, Sri Lanka, Turkey and United Arab Emirates [4].

Pigeonpea, is tolerant to various biotic and abiotic stresses including many strains of sterility mosaic, drought, salinity etc. thus has drawn the interest of plant research community to examine its biology. Plant stress responses are often regulated by multiple signalling pathways that activate gene transcription and associated downstream mechanisms [5, 6]. The cis-regulatory elements of related transcription factors (TFs) are the functional elements located in the promoter region of the genes that determine the spatial and temporal transcriptional activity of the gene during various biological processes [7].

In this study, we performed genome wide sequence analysis of pigeonpea for the identification of TF and developed a comprehensive database named as Pigeonpea Transcription Factors Database (PpTFDB) by using various computational analyses. We used our in-house data published in 2011 as the first draft of the pigeonpea genome sequence [1] for the development of PpTFDB. Transcription factors (TFs) are an essential part of the transcription machinery and the identification, characterization and expression analysis of transcription factor families is one of the major areas of research. Transcription factors are involved in the control of gene expression in all living organisms. Transcription factors (TFs) regulate the gene expression through binding to specific cis-regulatory sequences in the promoters of their target genes [8]. The control of gene expression in plants as well as in other living organisms is essential for the regulation of biological processes like development, differentiation and response to various environmental signals [9–11]. Many TF databases are available for many plant species whose data is available in the public domain. However, such transcription database is not available in case of pigeonpea. Therefore the objectives of present study were to construct a comprehensive Pigeonpea Transcription Factor Database (PpTFDB) which will serve as a central resource for researchers of legume community.

## Materials and methods

### Identification of transcription factors

In the pigeonpea genome sequence we predicted 47891 proteins coding genes along with their CDS by using *Glycine max* (soybean) as a reference for the gene prediction by FGENESH program. The whole genome sequence of pigeonpea was downloaded from https://www.ncbi.nlm.nih.gov/bioproject/258132. The complete set of TF sequences was downloaded from Plant Transcription Factor Database [12] and HMM profiles were created for each of the TF family by using the HMMER program [13]. The HMM profiles was then used to search against the pigeonpea proteome data using HMMER program with default E-value. The raw alignments data file was manually inspected to ensure reliability. A total of 1829 putative TFs were identified and characterized into 55 TF families (S1 Table). The complete set of sequence data of each TF family including amino acid, CDS and genomic DNA sequence is made available to the users and can be downloaded from PpTFDB for further analysis.

### Annotation of identified TFs

In order to incorporate complete information for the putative TFs, we performed annotations at gene, protein and family levels. The TF protein sequences were scanned by using standalone version of InterProScan program [14], which contains various inbuilt functional databases including Pfam (http://pfam.xfam.org/), PRINTS (http://www.bioinf.manchester.ac.uk/dbbrowser/PRINTS/), SMART (http://smart.embl-heidelberg.de/), PrositeProfiles (http://prosite.expasy.org/), PrositePatterns (http://prosite.expasy.org/), SUPERFAMILY (http://supfam.cs.bris.ac.uk/), Panther (http://www.pantherdb.org/panther/) [15] and gene ontology (GO) (http://amigo.geneontology.org/) to predict the functional domains and structural motifs along with their annotation details. To acquire the corresponding physical position of the TFs on individual chromosomes, the individual TF gene sequences were examined by local BLAST [16] search against the whole genome sequence of pigeonpea. The TFs properties like molecular weight, isoelectric point, aliphatic index, instability index and gravy index were calculated by ProtParam online server (http://web.expasy.org/protparam/) and the gene ontology (GO) were assigned to each of the putative TFs by using Blast2GO [17] program.

### Identification of SSR and orthologous groups

The CDS sequences of the putative TFs were used for SSRs (Simple Sequence Repeats) generation using MISA tool (http://pgrc.ipk-gatersleben.de/misa/). The identified SSRs were used to design primers by using BatchPrimer3 online tool (http://probes.pw.usda.gov/batchprimer3/) using various parameters (Range of primer length = 20–25 bp, Size of PCR product = 100–250 bp; with optimum of 280 bp, GC content of 40–60% with optimum of 50%). In order to find out the orthologous to each of the identified putative TFs, the protein sequences were analysed with protein BLAST [16] program against the protein sequences of various legume crops including soybean (*G*. *max*), mung bean (*Vigna radiata*), adzuki bean (*Vigna angularis*), common bean (*Phaseolus vulgaris*) and barrel medicago (*Medicago truncatula*) using default parameters. Homology with >80% similarity was considered as a significant threshold for selecting anorthologue (S2 Table).

### Database construction and implementation

**Fig 1:**
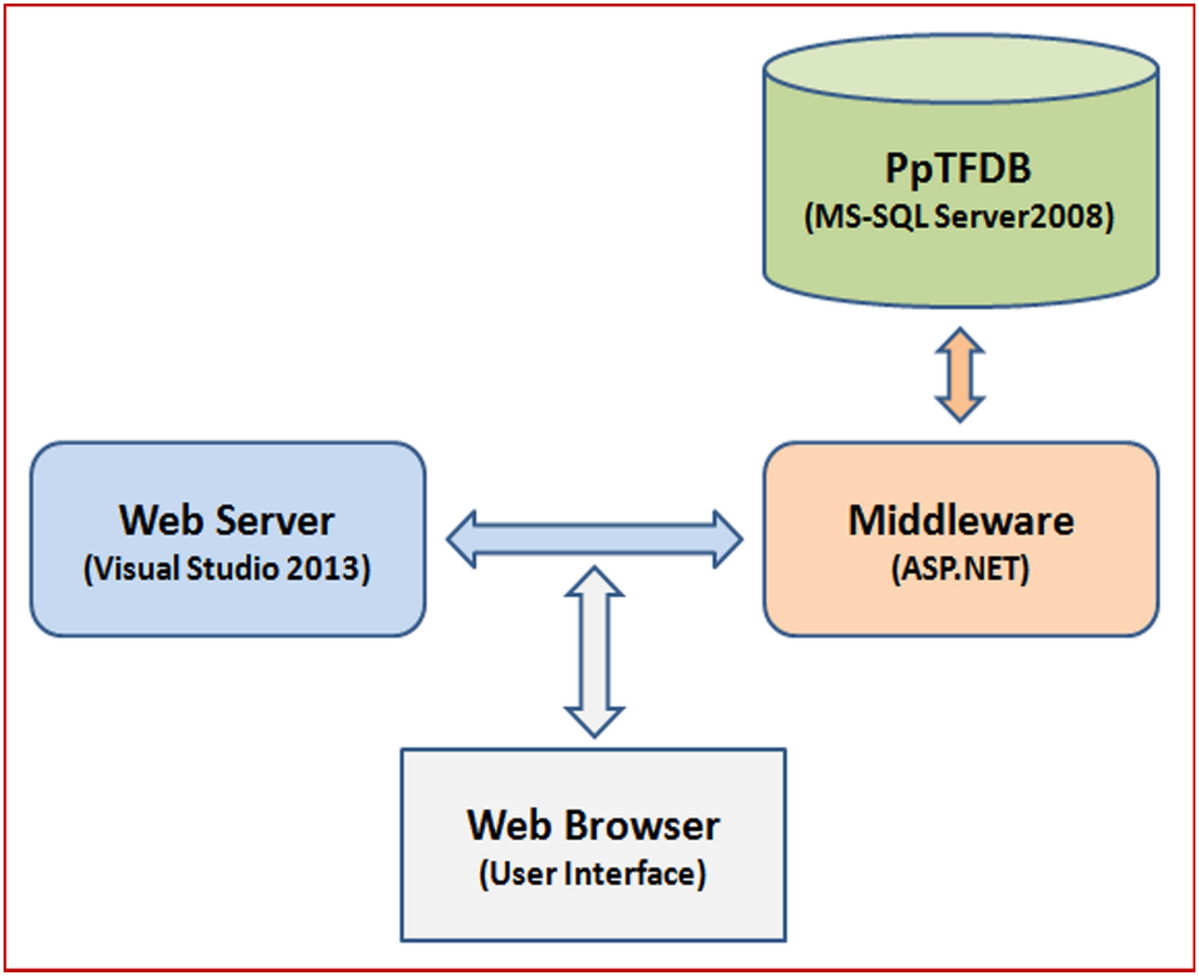
Three-Level Schema Architecture of PpTFDB.

**Fig 2:**
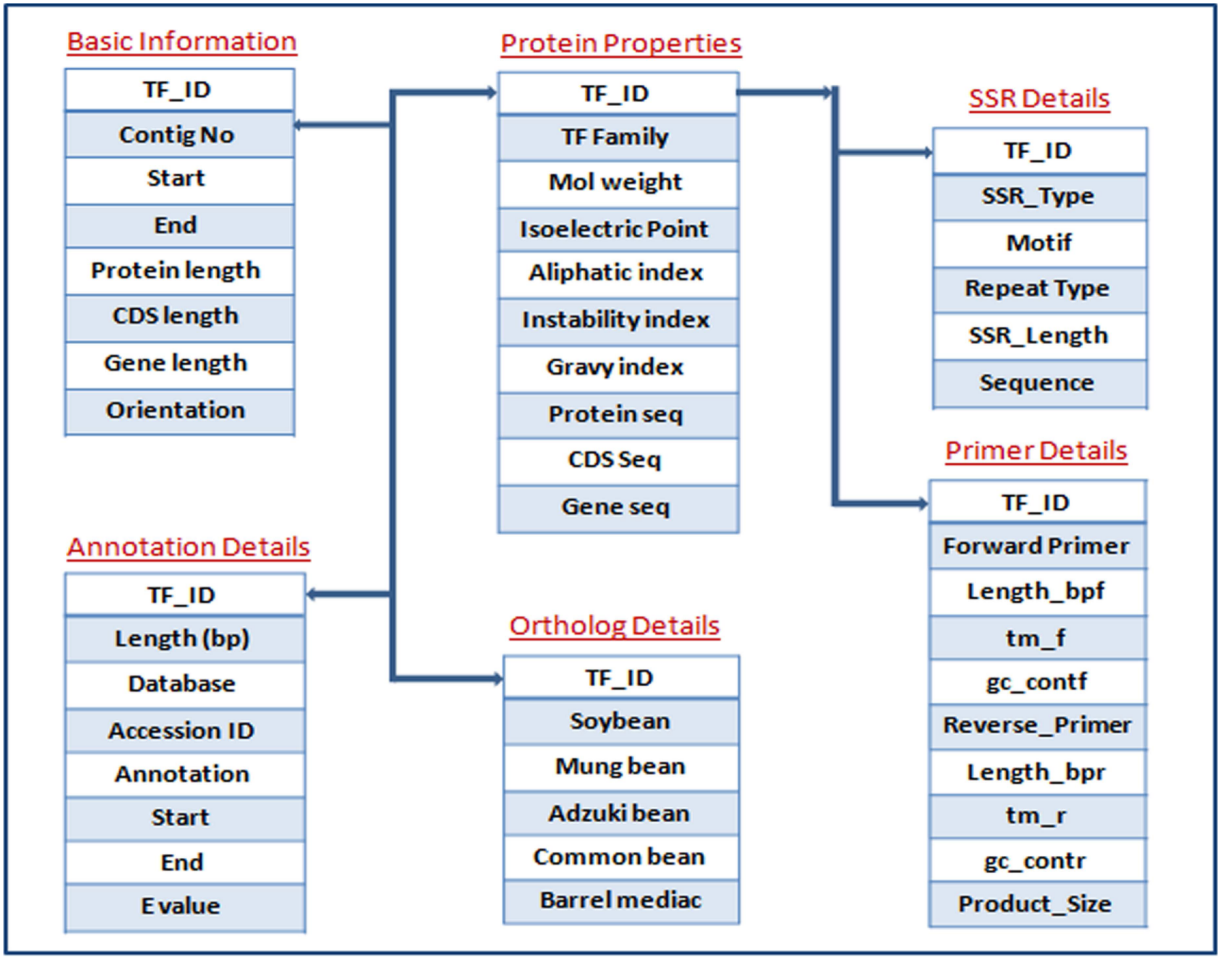
Database schema of PpTFDB. Schema showing different tables with their comprehensive unique key and data type information in each table available in PpTFDB.

The pigeonpea Transcription Factor database (PpTFDB) was designed by using Three-Level Schema Architecture (Fig 1). All the data tables were deposited in the MSSQL Server 2008 in relational manner for custom search and easy retrieval of data. A diagrammatic representation of data tables incorporated (database schema) in the PpTFDB is shown in Fig 2. A brief description about each TF family and a hyperlink to respective literature introduces precisely about respective TF family. The hyperlinks to the external databases such as Pfam (http://pfam.sanger.ac.uk/), SMART (http://smart.embl-heidelberg.de/), PrositeProfiles (http://prosite.expasy.org/), SUPERFAMILY (http://supfam.cs.bris.ac.uk/), Panther (http://www.pantherdb.org/panther/) and Gene Ontology (GO) (http://amigo.geneontology.org/) enables the user to go to the database and get comprehensive information about a candidate TFs.

## Results and discussion

### Database search criteria

In PpTFDB under the search tab, search by TF ID facilitates the user to search the particular transcription factor based on assigned TF ID to those TFs. After entering in the TF ID and click search button, the database will provide complete details of TF that includes chromosomal location, physical properties, annotation details, sequence information, orthologue details and SSR details with three pairs of primers, designed for each SSRs. Similarly user can also search the database by entering Pfam ID, SMART ID, InterProScan ID, ProSiteProfiles ID, SUPERFAMILY ID and Panther ID of interest (S1 Fig). After entering the function ID and search, users will be redirected to the page having list of TFs with annotation information and hyperlinked field’s accession id, detail information, SSR details and orthologs. By clicking on a particular accession id, the related database page will be opened which will have detailed information about the family. The “detail information” link redirects to the page containing information includes contig position, length, TF family, physico-chemical properties and sequence information. The SSR details link redirects to the page containing information of the predicted SSR in particular TFs and a link to the primer details that contains information on three pairs of primer. The orthologs link provides the information about percentage orthology with other legume crops.

#### Browse by TF categories

This browse option facilitates the user to search available transcription factors in PpTFDB according to their TF family. Total 1829 TFs were predicted and categorized into 55 TF families. This web page contains information about the predicted 55 TF families and the no. of TFs present in each family. For the detailed information about particular TF family click on ‘Get details’ button which will redirects the user to a separate web page. This page includes information about the TF family, PubMed link for literature, the list of TFs available under this category and hyperlinked fields detail information, annotation details, SSR details and orthologs details (S2 Fig). These hyperlink fields will provide various details about TFs like contig name in which the TFs is present, start- and end- position, lengths, orientation, physical properties, protein CDS and genomic sequence, functional annotation information predicted by InterProScan, predicted SSR information with three pairs of primers and percentage orthology with other legume crops.

#### Browse by GO categories

This browse option enables the user to search transcription factors in PpTFDB according to the assigned GO category. The predicted 1829 TFs were subjected to BLAST2GO program to assign their respective GO categories like cellular component, biological process and molecular function. This web page containing the information about the no. of TFs assigned to a particular GO category and a hyperlinked field GO id containing sequences in the fasta format are made available for the users for further analysis (S3 Fig).

The Pigeonpea genome sequenced by two different groups *Singh et al*. [1] and *Varshney et al*. [18] for the same genotype ICPL 87119, known as Asha. For developing Pigeonpea Transcription Factor Database (PpTFDB), we used in-house Pigeonpea data sequenced by *Singh et al*. [1] because it is available in the form of contigs assigned to specific chromosome. The difference in no. of transcription factors listed in Plant Transcription Factor Database (http://planttfdb.cbi.pku.edu.cn/index.php?sp=Cca) for each family and our PpTFDB is due to the differences in the coverage of genome size. *Singh et al*. [1] sequenced about 60% of the estimated 858 Mb size of the pigeonpea genome whereas *Varshney et al*. [18] covered 72.7% of the estimated genome size. Hence, variation in number of TF identified in present study and that of present in PpTFDB might be due to the availability of 60–72% of the genome sequence data available in the public domain. Once the whole genome sequence data is generated and made available, the present database will be further enriched with the updated information. The sequenced plant genomes data available in public domain enables the researchers to carryout high-throughput research in the area of comparative genomics and transcriptomic data analysis [19], gene expression analysis [20], functional genomics [21] proteomics [22] and database development [23]. Such databases are very helpful for the biologists for functional validations of the genes identified *in silico*.

Transcription factors (TFs) play a major role in controlling various processes like responses to biotic and abiotic stresses, development, differentiation, metabolism and defense responses to pathogens etc. TFs also play roles in plant innate immunity by regulating genes related to pathogen-associated molecular pattern-triggered immunity, effector-triggered immunity, hormone signaling pathways and phytoalexin synthesis [24, 25]. Recently, the structure-based approaches of TF-binding site prediction have gained substantial interest due to the rapidly increasing structural database of TFs-DNA complexes that can provide much more information for the prediction of TF binding sites than sequence-based approaches. Most of the structure-based approaches have been used as a model that is based on solved TF-DNA complexes and a scoring function for evaluating the binding affinity between a DNA subsequence and a transcription factor [26]. The integration of genomics information with the knowledge obtained from functional and structural studies will facilitate better understanding of gene regulation in plants for the development of new varieties with agronomically important traits, and regulation of plant defense mechanisms.

We believe that the PpTFDB will be beneficial for researchers as well as plant breeders who are working for the improvement of legume crops and genome-wide studies of TF families. This database is user friendly and also provides the researchers options to freely download the entire data set used to build this database.

## Conclusion

PpTFDB is a user-friendly web interface that provides a range of information about the pigeonpea TFs for public domain. This database will decrease the effort in extracting genomic information about pigeonpea TF families by the researchers and breeders. The availability of the comprehensive information, including individual or family-wise TFs, protein functional annotation and gene ontology annotation of predicted TFs in the database is expected to prioritize the functional analysis of TFs of interest. We believe that the information about pigeonpea TFs available in the database will support basic and applied research. The database will be updated on regular basis with the availability of updated version of data. Further, additional information related to the pigeonpea TFs, and gene expression related data including expression patterns in different cultivars and genomic variations will also be integrated in the database in near future.

## Supporting information

S1 Fig Shows different search options available in PpTFDB. Search criteria’s including search by TF ID, protein functional IDs and search results shown by flow diagram.
(TIF)

S2 Fig Shows TF categories family wise available in PpTFDB. TFs categories along with their respective TFs no. present in each family with detail information.
(TIF)

S3 Fig Shows TFs available in PpTFDB with their respective GO categories. (TIF)

S1 Table List of predicted TFs family wise with their contig information. (XLSX)

S2 Table List of TFs with their orthology predicted in different legume crops. (XLSX)

## References

[1] Singh NK, Gupta DK, Jayaswal PK, Mahato AK, Dutta S, Singh S, et al The first draft of the pigeonpea genome sequence. J. Plant Biochem. Biotechnol. 2012; 21:98 doi: 10.1007/s13562-011-0088-8 24431589 10.1007/s13562-011-0088-8 PMC3886394

[2] Satheesh V, Jagannadham PTK, Chidambaranathan P, Jain PK, Srinivasan R. NAC transcription factor genes: genome-wide identification, phylogenetic, motif and cis-regulatory element analysis in pigeonpea (*Cajanus cajan* (L.) Millsp.). Mol. Biol. Rep. 2014; 41:7763–7773. doi: 10.1007/s11033-014-3669-5 25108674 10.1007/s11033-014-3669-5

[3] Bhawna, Bonthala VS, Gajula MNVP. PvTFDB: a Phaseolus vulgaris transcription factors database for expediting functional genomics in legumes. Database (Oxford). 2016; doi: 10.1093/database/baw114 27465131 10.1093/database/baw114 PMC4962766

[4] FAOSTAT. http://faostat.fao.org/ (accessed on 16 November 2016).

[5] Varshney RK, Penmetsa RV, Dutta S, Kulwal PL, Saxena RK, Sharma TR. et al Pigeonpea genomics initiative (PGI): an international effort to improve crop productivity of pigeonpea (*Cajanus cajan* L.). Mol. Breeding. 2010; 26:393–408. doi: 10.1007/s11032-009-9327-2 20976284 10.1007/s11032-009-9327-2 PMC2948155

[6] Priyanka B, Sekha K, Sunita T, Reddy VD, Rao KV. Characterization of expressed sequence tags (ESTs) of pigeonpea (*Cajanus cajan* L.) and functional validation of selected genes for abiotic stress tolerance in Arabidopsis thaliana. Mol. Genet. Genomics. 2010; 283:273–287. doi: 10.1007/s00438-010-0516-9 20131066 10.1007/s00438-010-0516-9

[7] Sirhindi G, Sharma P, Arya P, Goel P, Kumar G, Acharya V. et al Genome-wide characterization and expression profiling of TIFY gene family in pigeonpea (*Cajanus cajan* (L.) Millsp.) Under copper stress. J. Plant Biochem. Biotechnol. 2015; 25:301–310. doi: 10.1007/s13562-015-0342-6

[8] Franco-Zorrilla JM, Lopez-Vidriero I, Carrasco JL, Godoy M, Vera P, Solano R. DNA-binding specificities of plant transcription factors and their potential to define target genes. Proceedings of the National Academy of Sci. 2014; 111:2367–2372. doi: 10.1073/pnas.1316278111 24477691 10.1073/pnas.1316278111 PMC3926073

[9] Yanagisawa S. Transcription factors in plants: Physiological functions and regulation of expression. Journal of Plant Res. 1998; 111:363–371. doi: 10.1007/BF02507800

[10] Malviya N, Gupta S, Singh VK, Yadav MK, Bisht NC, Sarangi BK, et al Genome wide in silico characterization of Dof gene families of pigeonpea (*Cajanus cajan* (L) Millsp.). Mol. Biology Rep. 2014; 42:535–552. doi: 10.1007/s11033-014-3797-y 25344821 10.1007/s11033-014-3797-y

[11] Agarwal G, Garg V, Kudapa H, Doddamani D, Pazhamala LT, Khan AW.et al Genome-Wide Dissection Of AP2/ERF And HSP90 Gene Families In Five Legumes And Expression Profiles In Chickpea And Pigeonpea. Plant Biotechnology Jour. 2016; 14:1–15. doi: 10.1111/pbi.12520 26800652 10.1111/pbi.12520 PMC5066796

[12] Jinpu J, Feng T, De-Chang Y, Yu-Qi M, Lei K, Jingchu L. et al PlantTFDB 4.0: toward a central hub for transcription factors and regulatory interactions in plants. Nucleic Acids Res. 2016; doi: 10.1093/nar/gkw982 27924042 10.1093/nar/gkw982 PMC5210657

[13] Eddy SR. Profile of hidden Markov models. Bioinformatics. 1998; 14:755–763. 9918945 10.1093/bioinformatics/14.9.755

[14] Zdobnov EM, Apweiler R. InterProScan—an integration platform for the signature-recognition methods in InterPro. Bioinformatics. 2001; 17: 847–848. 11590104 10.1093/bioinformatics/17.9.847

[15] Huaiyu M, Sagar P, Anushya M, John TC, Paul DT. PANTHER version 10: expanded protein families, functions and analysis tools. Nucleic Acids Res. 2015; 44: D336–D342. doi: 10.1093/nar/gkv1194 26578592 10.1093/nar/gkv1194 PMC4702852

[16] Altschul SF, Gish W, Miller W. Myers EW, Lipman DJ. Basic Local Alignment Search Tool. Journal of Mol. Bio.1990;215: 403–410. doi: 10.1016/S0022-2836(05)80360-2 2231712 10.1016/S0022-2836(05)80360-2

[17] Conesa A, Gotz S, Garcia-Gomez JM, Terol J, Talon M, Robles M. Blast2GO: a universal tool for annotation, visualization and analysis in functional genomics research. Bioinformatics. 2005;21: 3674–3676. doi: 10.1093/bioinformatics/bti610 16081474 10.1093/bioinformatics/bti610

[18] Varshney RK, Chen W, Li Y, Bharti AK, Saxena RK, Schlueter JA. et al Draft genome sequence of Pigeonpea (*Cajanus cajan*), an orphan legume crop of resource-poor farmers. Nature Biotechnology. 2012; 30:83–89. doi: 10.1038/nbt.2022 22057054 10.1038/nbt.2022

[19] Qiao Z, Pingault L, Nourbakhsh-Rey M, Libault M. Comprehensive Comparative Genomic and Transcriptomic Analyses of the Legume Genes Controlling the Nodulation Process. Front. Plant Sci. 2016; 7:34 doi: 10.3389/fpls.2016.00034 26858743 10.3389/fpls.2016.00034 PMC4732000

[20] Garg R, Kumari R, Tiwari S, Goyal S. Genomic Survey, Gene Expression Analysis and Structural Modeling Suggest Diverse Roles of DNA Methyltransferases in Legumes. PLoS ONE. 2014;9:e88947 doi: 10.1371/journal.pone.0088947 24586452 10.1371/journal.pone.0088947 PMC3934875

[21] Maibam A, Tyagi A, Satheesh V, Mahato AK, Jain N, Raje RS.et al Genome-wide identification and characterization of heat shock factor genes from pigeonpea (*Cajanus cajan*). Mol. Plant Breed. 2015; 6:1–11. doi: 10.5376/mpb.2015.06.0007

[22] Rathi D, Gayen D, Gayali S, Chakraborty S, Chakraborty N. Legume proteomics: Progress, prospects, and challenges. Proteomics. 2015; 16:310–327. doi: 10.1002/pmic.201500257 26563903 10.1002/pmic.201500257

[23] Dash S, Campbell JD, Cannon EKS, Cleary AM, Huang W, Kalberer SR. et al Legume information system (LegumeInfo.org): a key component of a set of federated data resources for the legume family. Nucleic Acids Res.2015; 44:D1181–D1188. doi: 10.1093/nar/gkv1159 26546515 10.1093/nar/gkv1159 PMC4702835

[24] Ambawat S, Sharma P, Yadav NR, Yadav RC. MYB transcription factor genes as regulators for plant responses: an overview. Physiol. Mol. Biol. Plants.2013; 19(3):307–21. doi: 10.1007/s12298-013-0179-1 24431500 10.1007/s12298-013-0179-1 PMC3715649

[25] Seo E and Choi D. Functional studies of transcription factors involved in plant defenses in the genomics era. Briefings in Functional Genomics. 2015; 14(4):260–267. doi: 10.1093/bfgp/elv011 25839837 10.1093/bfgp/elv011

[26] Liu Z, Guo JT, Li T, Xu Y. Structure-based prediction of transcription factor binding sites using a protein-DNA docking approach. Proteins. 2008; 72:1114–1124. doi: 10.1002/prot.22002 18320590 10.1002/prot.22002

