# Supplementary figures and images for: PpTFDB: A pigeonpea transcription factor database for exploring functional genomics in legumes
Source: PLoS One. 2017 Jun 26;12(6):e0179736. doi: 10.1371/journal.pone.0179736 (PMC5484553; doi:10.1371/journal.pone.0179736)

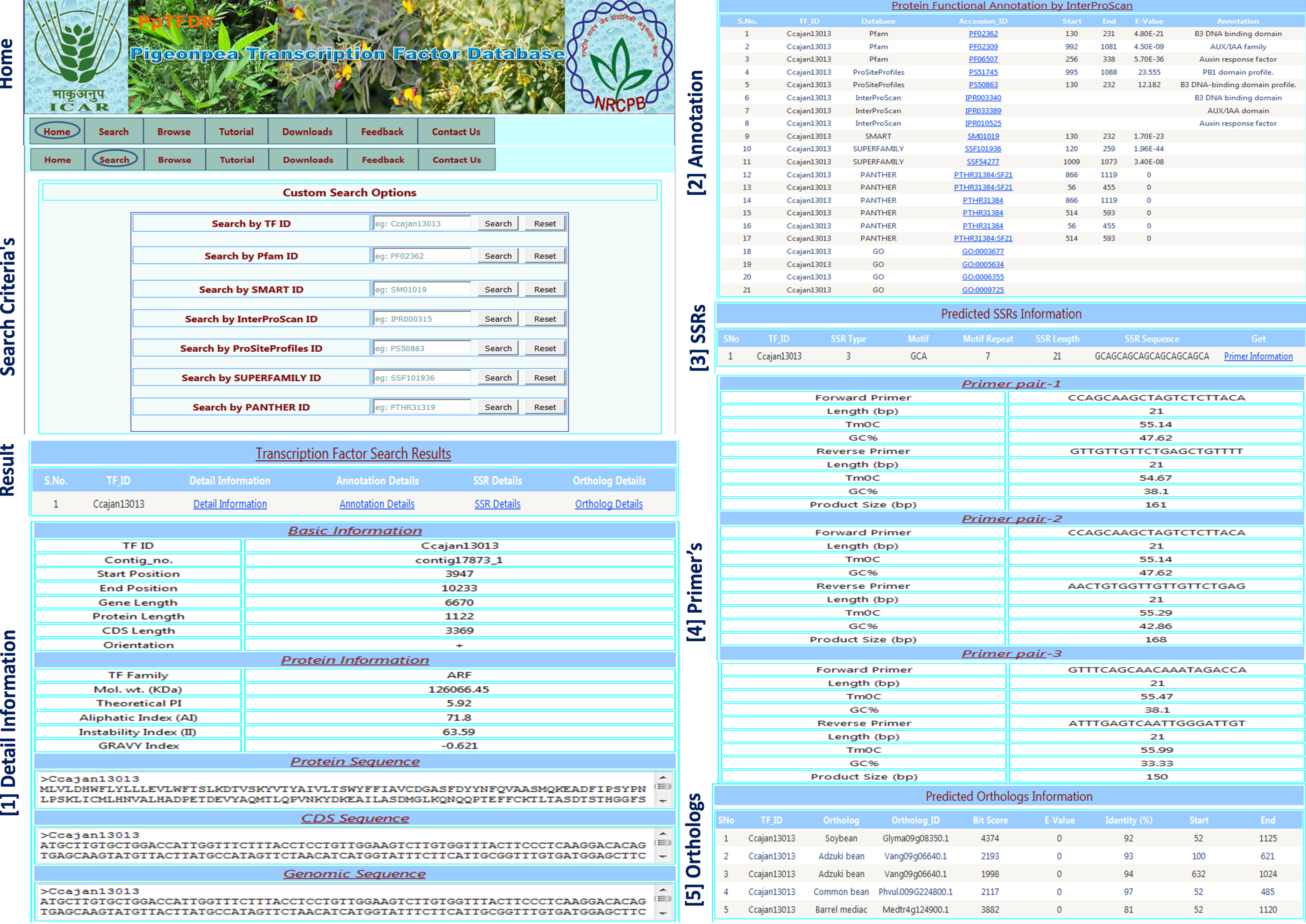

Supplement: S1 Fig — Search criteria’s including search by TF ID, protein functional IDs and search results shown by flow diagram. (TIF) [file pone.0179736.s001.tif]

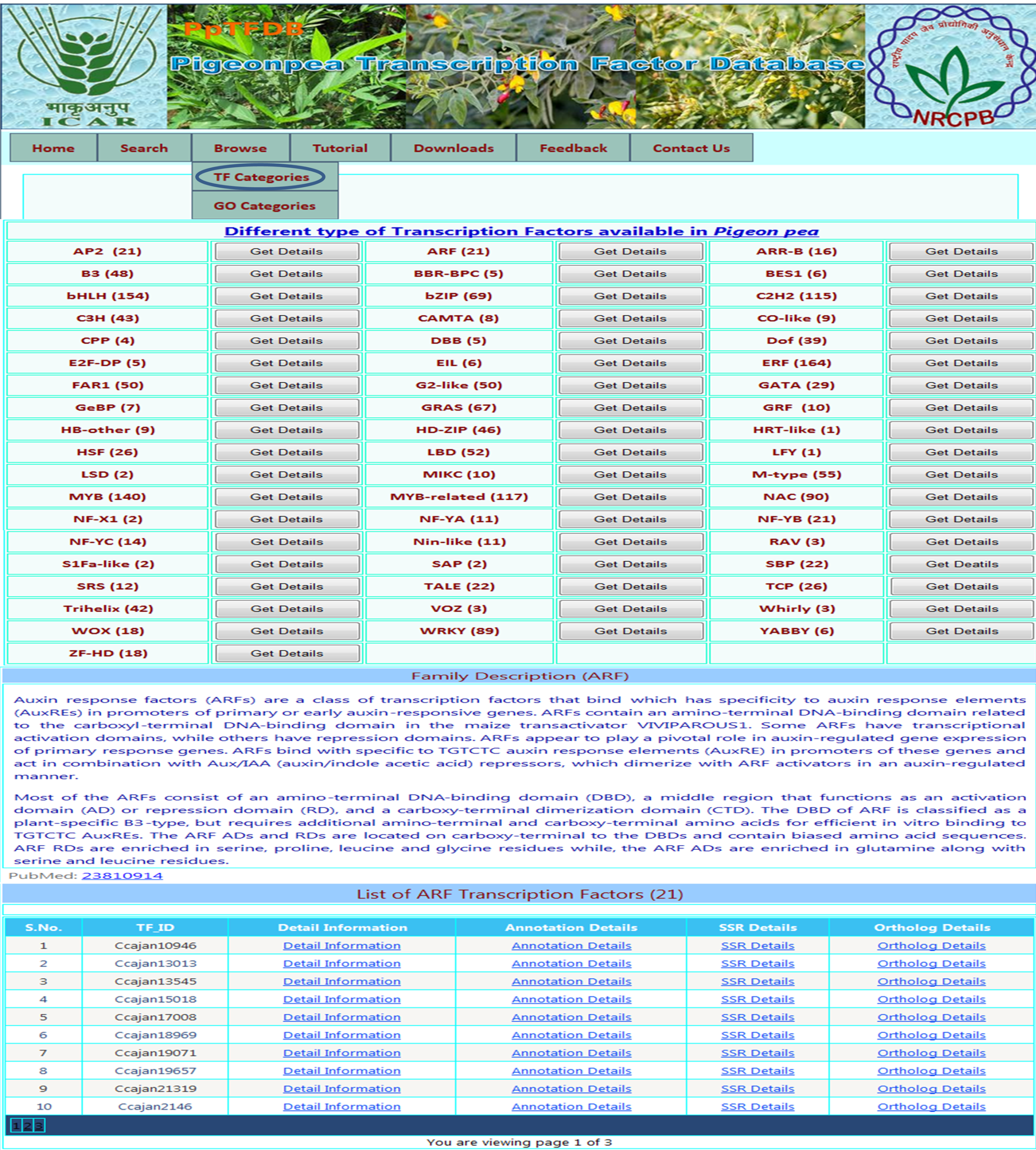

Supplement: S2 Fig — TFs categories along with their respective TFs no. present in each family with detail information. (TIF) [file pone.0179736.s002.tif]

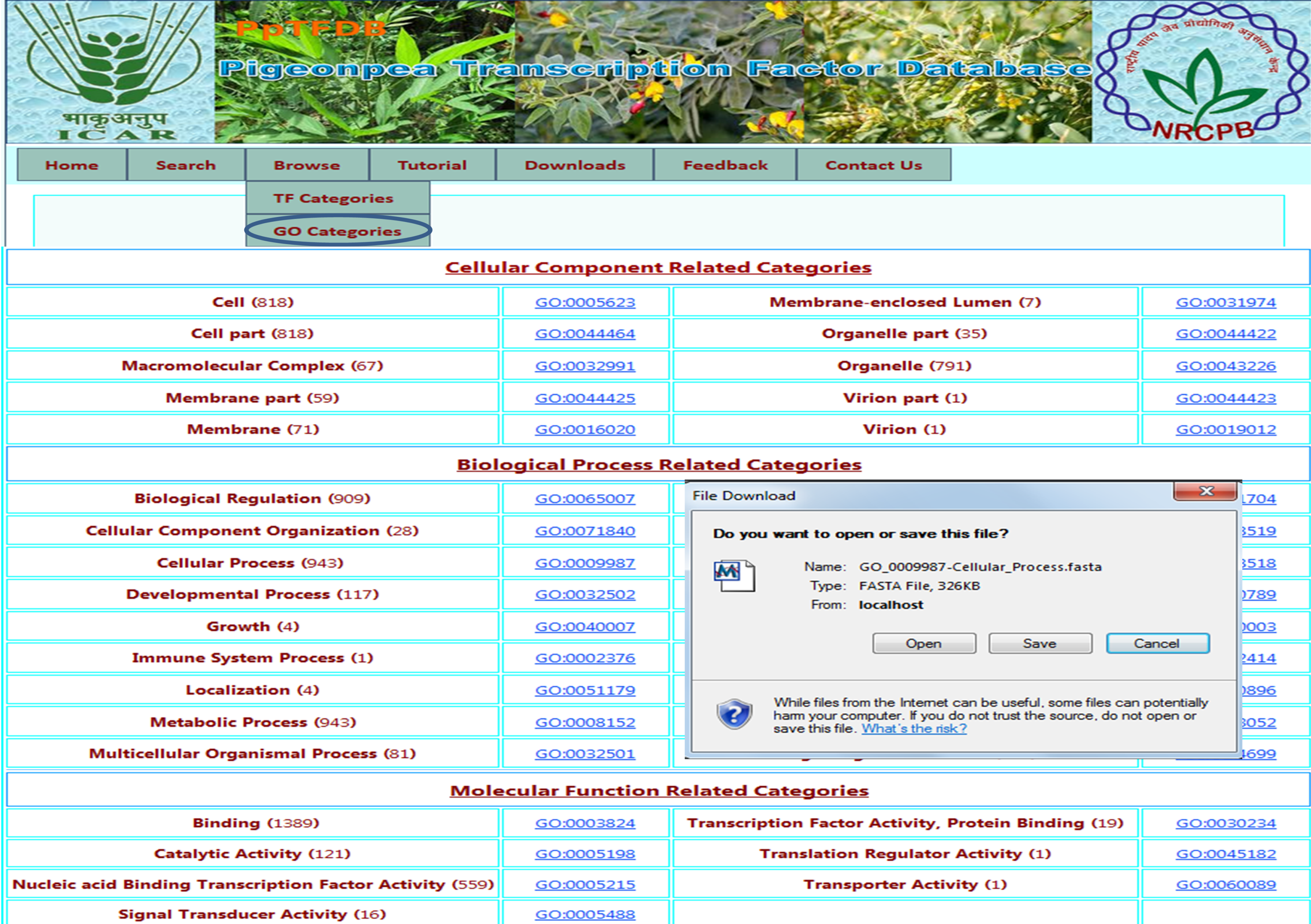

Supplement: S3 Fig — (TIF) [file pone.0179736.s003.tif]
